# Influenza Virus A (H1N1) in Giant Anteaters (*Myrmecophaga tridactyla*)

**DOI:** 10.3201/eid1507.081574

**Published:** 2009-07

**Authors:** Sally Nofs, Mohamed Abd-Eldaim, Kathy V. Thomas, David Toplon, Dawn Rouse, Melissa Kennedy

**Affiliations:** Nashville Zoo at Grassmere, Nashville, Tennessee, USA (S. Nofs, D. Toplon, D. Rouse); University of Tennessee College of Veterinary Medicine, Knoxville, Tennessee, USA (M. Abd-Eldaim, K. Thomas, M. Kennedy); Suez Canal University Faculty of Veterinary Medicine, Ismailia, Egypt (M. Abd-Eldaim); 1 These authors contributed equally to this article.

**Keywords:** Influenza, H1N1 subtype, viruses, anteaters, Myrmecophaga tridactyla, zoonosis, dispatch

## Abstract

In February 2007, an outbreak of respiratory disease occurred in a group of giant anteaters (*Myrmecophaga tridactyla*) at the Nashville Zoo. Isolates from 2 affected animals were identified in March 2007 as a type A influenza virus related to human influenza subtype H1N1**.**

Emergence of viruses in new hosts is a continuing concern in public health surveillance and thus is the focus of intense research. The ability to move among different species may enable mutations and changes in phenotypes as the virus adapts to a new host (*1*). Influenza virus is a prime example of a pathogen with the ability to infect not only its avian reservoirs but also mammalian species such as swine, horses, dogs, cats, ferrets, whales, and humans.

In March 2007, we documented the occurrence of influenza in giant anteaters (*Myrmecophaga tridactyla*). Giant anteaters are indigenous to neotropical regions of Central and South America, and, although they are extinct or endangered in some regions, overall their status is listed as near threatened by the International Union for Conservation of Nature (*2*,*3*).

## The Study

A colony of 11 adult anteaters (7 males, 4 females) and 1 neonate was housed at the Nashville Zoo at Grassmere, Nashville, Tennessee, USA. The colony experienced an outbreak of respiratory disease beginning in February 2007. The anteaters were housed separately in stalls in the same building with shared ventilation, with the exception of the nursing neonate who was housed with his dam. There was no contact with animals outside the colony. The primary caretaker of the colony had no contact with other animals housed at the zoo. No other species experienced respiratory disease at the zoo during the outbreak. Only the primary caretaker had sustained direct contact with any members of the colony.

The index case occurred on February 8 in 1 animal that was being treated twice a day for a superficial wound. The respiratory disease was characterized clinically by severe nasal discharge and congestion, inappetence, and lethargy. Within several days, all adult animals in the colony were affected. Only the neonate appeared to remain unaffected. The caretakers overseeing the colony, with the exception of the attending veterinarian, were also ill with respiratory disease, including the primary caretaker. The onset of the caretakers’ illness coincided with the illness in the anteaters. No diagnostic testing was conducted for the caretakers during the outbreak.

Nasal discharge samples were collected on February 15 for virus isolation from 3 animals, including the index case. These samples were shipped overnight on cold packs to the Clinical Virology Laboratory at the University of Tennessee College of Veterinary Medicine. These samples were prepared for virus isolation in Dulbecco minimal essential medium supplemented with antimicrobial drugs, antifungals, and 5% fetal bovine serum (Atlanta Biologicals, Norcross, GA, USA; and Cambrex Bioscience Walkersville, Walkersville, MD, USA). Cell lines injected included Vero, rabbit kidney, MDCK, and Crandell-Reese feline kidney (American Type Culture Collection, Manassas, VA, USA). Cytopathic effects were noted in the MDCK cell lines on first passage in 2 of the 3 isolations; no cytopathic effects were noted in the isolation from the third sample.

Cell culture supernatant from the infected flasks was tested for hemagglutination by using 10% guinea pig erythrocytes, and agglutination was observed. Virus-infected cell pellets were prepared for electron microscopic examination (*4*). Examination showed an enveloped virus of 100–120 nm diameter (www.vet.utk.edu/pubs/mkenned2/EID_08-1574). Slides of infected cells were also prepared and examined by direct immunofluorescent antibody (IFA) assay by using influenza A- and B-specific antiserum (Diagnostic Hybrids, Athens, OH, USA); positive fluorescence was observed only with type A-specific antiserum. Based on the morphology and IFA results identifying a type A influenza virus, reverse transcription and PCR were conducted on the isolate from one of the animals by using type A-specific primers encompassing the entire hemagglutinin (HA), neuraminidase (NA), nonstructural protein 1 (NS1), and RNA polymerase 2 (PB2) gene segments as previously described (*5*). Nucleotide sequencing of HA, NA, PB2, and NS1 gene amplification products were conducted by Molecular Biology Resources (University of Tennessee, Knoxville, TN, USA) using an ABI prism dye terminator cycle sequencing reaction kit and an ABI 373 DNA sequencer (Perkin Elmer, Foster City, CA, USA) (Genbank accession nos. EU543278, EU543279, FJ478393, and FJ785200, respectively). High nucleotide identity (>99%) was found between the anteater isolate and human influenza virus isolate type A strain Tennessee/UR06-0119/2007(H1N1) and others from the United States isolated in 2006 and 2007 (data not shown). Phylogenetic analysis (MegAlign program with ClustalW align, Lasergene package; DNAStar, Madison, WI, USA) of the HA and NA amplification products indicated a close relationship among these isolates (Figure).

**Figure:**
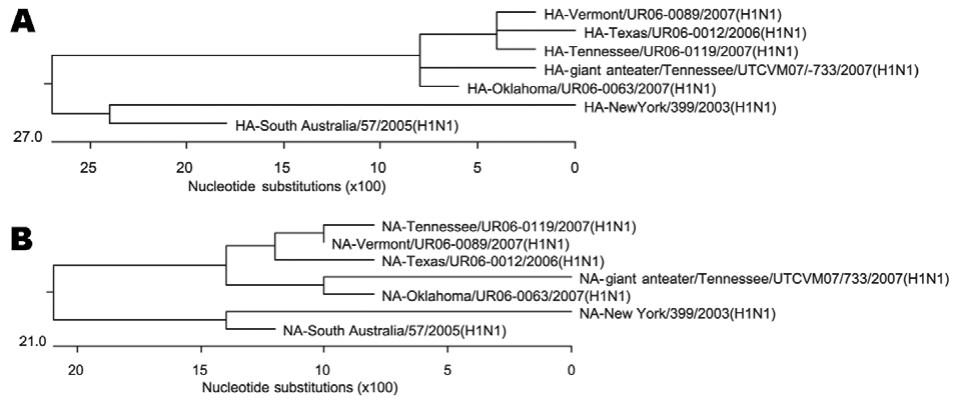
Phylogenetic tree based on the nucleotide sequence of the A) hemagglutinin (HA) gene and B) neuraminidase (NA) gene of the anteater isolate of influenza virus with 6 related isolates obtained from GenBank. GenBank accession numbers for the other isolates used: Tennessee/UR06-0119/2007(H1N1): HA-CY027379, NA-CY027381; Texas/ur06/0012/2006(H1N1): HA-CY025213, NA-CY025215; Vermont/UR06/0089/2007(H1N1): HA-CY025795; NA-CY025797; Oklahoma/UR06/0063/2007(H1N1): HA-CY027771, NA-CY027773; New York/399/2003(H1N1): HA-CY002808, NA-CY002810; and South Australia/67/2005(H1N1): HA-CY016691, NA-CY016693.

Serologic analysis was not feasible at the time of the outbreak due to the need to use anesthesia to immobilize the animals for blood collection. However, samples collected from 3 animals in months following the outbreak were made available for serologic testing, as well as samples from these same animals collected at various times prior to the outbreak. The samples had been stored at –20°C. Testing was conducted by hemagglutination inhibition using the anteater influenza isolate (*6*). The serologic results are shown in the Table. Evidence of seroconversion was observed in 2 of the animals, and 1 animal had evidence of infection prior to the 2007 outbreak.

**Table:** Serologic results on samples collected from 3 anteaters before and after the February 2007 respiratory disease outbreak at the Nashville Zoo at Grassmere, Nashville, Tennessee, USA*

Animal no.	Before outbreak			After outbreak		Virus isolation
	Date collected	HI titer		Date collected	HI titer	
1	2004 Apr	0		2007 Aug	32	No sample
2	2006 Aug	4		2008 Jun	64	No sample
3	2006 Mar	32		2008 Oct	64	Virus isolated

*HI, hemagglutination inhibition.

## Conclusions

This respiratory disease outbreak among members of an anteater colony was caused by an influenza virus isolate closely related to the human influenza virus (H1N1) strains circulating in the concurrent year. Serologic analysis of samples from animals involved in the outbreak confirmed infection; seroconversion was documented in 2 animals, and 1 animal appeared to have been exposed to and infected with influenza virus a year before the described outbreak. This animal had been imported as a juvenile in 2003 and was ill at that time with a respiratory disease. It is not known what the cause of this earlier illness was or if exposure to influenza in the intervening years had occurred. Analysis of 4 viral genes sequenced indicated high homology with the human influenza virus (H1N1) isolates.

The differences between the anteater isolate and circulating human strains did not occur at sites known to be antigenically or functionally important; thus, these minor changes do not appear to alter the antigenicity or the function of the encoded proteins (*7*, E. Gorvokova, pers. comm.). We concluded that, based on the genetic sequence of the virus isolated from the anteaters and on the fact that the colony was not exposed to animals other than the human caretakers, the caretakers were the most likely source of the virus affecting the anteater colony. Further genetic sequencing will be required to determine if this interspecies transmission arose as a result of mutations of the virus, including reassortment.

Influenza is known to cross species lines into mammalian species. Birds, in particular waterfowl (Family Anatidae), are the natural reservoir for influenza viruses. Reassortment between avian and mammalian strains may occur in swine because they express receptors for avian and mammalian influenza (*8*). An entire influenza virus may undergo interspecies transmission without reassortment. Two recent notable incidences of this are the transmission of equine influenza virus (H3N8) to dogs (*9*) and the transmission of avian influenza virus (H5N1) to humans as well as to various carnivore species (*10*–*13*). With the first mentioned, efficient dog-to-dog transmission was documented. For avian influenza virus (H5N1), sustained human-to-human transmission has not occurred. It is not clear whether animal-to-animal spread of this influenza virus occurred within the anteater colony or whether all affected animals were infected directly from the caretakers.

The isolation of influenza virus in giant anteaters highlights the difficulty of influenza surveillance as the full spectrum of mammalian hosts for influenza virus remains unknown. This host variability could potentially impact human populations as possible sources of zoonotic spread of influenza.
